# Whole genome sequencing and analysis of *Armillaria gallica* Jzi34 symbiotic with *Gastrodia elata*

**DOI:** 10.1186/s12864-023-09384-4

**Published:** 2023-05-23

**Authors:** Jinlong Cai, Ikram Muhammad, Bilian Chen, Peng Xu, Yiguo Li, Huini Xu, Kunzhi Li

**Affiliations:** Faculty of Life Science and Technology, Kunming University of Science and Technology, 650500 Kunming, China

**Keywords:** *Armillaria*, *Gastrodia elata*, Symbionts, Genome, Molecular mechanism

## Abstract

**Background:**

*Armillaria* species are plant pathogens, but a few *Armillaria* species can establish a symbiotic relationship with *Gastrodia elata*, a rootless and leafless orchid, that is used as a Chinese herbal medicine. *Armillaria* is a nutrient source for the growth of *G. elata*. However, there are few reports on the molecular mechanism of symbiosis between *Armillaria species* and *G. elata.* The genome sequencing and analysis of *Armillaria* symbiotic with *G. elata* would provide genomic information for further studying the molecular mechanism of symbiosis.

**Results:**

The de novo genome assembly was performed with the PacBio Sequel platform and Illumina NovaSeq PE150 for the *A. gallica* Jzi34 strain, which was symbiotic with *G. elata*. Its genome assembly contained ~ 79.9 Mbp and consisted of 60 contigs with an N50 of 2,535,910 bp. There were only 4.1% repetitive sequences in the genome assembly. Functional annotation analysis revealed a total of 16,280 protein coding genes. Compared with the other five genomes of *Armillaria*, the carbohydrate enzyme gene family of the genome was significantly contracted, while it had the largest set of glycosyl transferase (GT) genes. It also had an expansion of auxiliary activity enzymes AA3-2 gene subfamily and cytochrome P450 genes. The synteny analysis result of P450 genes reveals that the evolutionary relationship of P450 proteins between *A. gallica* Jzi34 and other four *Armillaria* was complex.

**Conclusions:**

These characteristics may be beneficial for establishing a symbiotic relationship with *G. elata*. These results explore the characteristics of *A. gallica* Jzi34 from a genomic perspective and provide an important genomic resource for further detailed study of *Armillaria*. This will help to further study the symbiotic mechanism between *A. gallica* and *G. elata*.

**Supplementary Information:**

The online version contains supplementary material available at 10.1186/s12864-023-09384-4.

## Background

*G. elata*, a famous Chinese herbal medicine used for more than 2000 years, mainly treats dizziness, headache, migraine, rheumatism, neuralgia and paralysis [1–3]. *G. elata* is a rootless and leafless orchid that is completely heterotrophic and dependent on fungi for nutrition. During the germination of *G. elata* seeds, they are infected by *Mycena* strains, become symbiotic with them, and then digest the infected fungi to nourish themselves, so that *G. elata* seeds sprout and grow into protocorms. As the protocorm of *G. elata* grows, it needs to be symbiotic with *Armillaria* strains, and digests them as nutrition to promote the further expansion and growth of *G. elata* tuber and complete the life cycle [4–6]. *G. elata* depends on *Armillaria* for nutrition in most periods. The key to successful artificial cultivation of *G. elata* is whether there are abundant sources of *Armillaria*. Different *Armillaria* strains affect the yield and quality of *G. elata* [7, 8]. Therefore, in the field of *G. elata* research, the symbiotic molecular mechanism between *G. elata* and *A. mellea* has attracted extensive attention.

The symbiotic mechanism between *G. elata* and *Armillaria* has rarely been revealed from the perspective of *Armillaria*, and most studies have focused on *G. elata*. Previous *G. elata* genomic analyses indicated an expansion of genes for novel metabolic processes and mycorrhizal association [9, 10]. Yuan et al. [9] found that strigolactone could stimulate the hyphal branching and development of *A. mellea*. *G. elata* may preferentially guide the colonization of *Armillaria* in its cortex through the ABC transporter mediating the secretion of strigolactone to the extracellular space. In addition, some antibacterial components (e.g., phytoalexin gastrodin and *Gastrodia* antifungal protein) are secreted in the cortex to prevent *Armillaria* overgrowth in the tubers and cause a disease. Transcriptome analysis of the symbiosis between *G. elata* and *A. mellea* showed that the symbiotic *G. elata* synthesizes organic nutrients and energy through the digestion of the invaded *A. mella* for the growth of the tuber [11]. Sucrose has been suggested to be transported from the fungus to *G. elata* as a carbon source, because high concentrations of sucrose and two sucrose transporter-like genes are highly expressed in young *Armillaria*-colonized tubers [12].

*Armillaria* species are pathogens causing root white rot disease [13, 14], but a few *Armillaria* species can establish a symbiotic relationship with *G. elata*. Previous studies have shown that the growth of *Armillaria* is closely related to the activities of extracellular enzymes, it secretes, such as laccase, cellulase, xylanase, pectinase and amylase. Extracellular enzymes play a decisive role in the ability of wood rot fungi to degrade nutrients, and their species and activity are related to the species of fungi [15]. The secretion of these extracellular enzymes provides a material basis for *Armillaria* to infect the epidermis of *G. elata*. It was also found that different extracellular enzymes play different roles in *Armillaria* growth. Laccase can degrade lignin and phenols, and its activity affects the ability of *Armillaria* to degrade lignin [16, 17]. Cellulase hydrolyzes cellulose to produce glucose, which can provide carbon source for the growth of the strain [15, 18]. Xylanase can decompose the polysaccharide structure xylan located in the secondary wall of plants [19]. Amylase mainly hydrolyses plant polysaccharide starch to provide nutrition. Pectinase can degrade pectin in plant cell stroma and primary cell walls [16, 19]. The study of the extracellular enzyme activity of *Armillaria* is of great significance for the growth of *Armillaria* and *G. elata*.

The pathogenicity and preferential saprophytic characteristics of *Armillaria* may affect its symbiotic relationship with *G. elata*. The virulence tests on the five *Armillaria* species show that *A. tabescens* has the weakest virulence, and *A. mellea* has the strongest virulence, followed by *A. ostoyae*, *A. gallica* and *A. cepistipes* [16, 20, 21]. Weakly pathogenic and preferentially saprotrophic *Armillaria* (e.g., *A. gallica*, and *A. cepistipes*) may readily establish a symbiotic relationship with *G. elata* [4]. However, *A. mellea* can also establish a symbiotic relationship with *G. elata*. This may be due to variation in virulence within a species; intraspecific variation has been found among strains of some *Armillaria* species (e.g., *A. ostoyae*) [16, 22]. Therefore, it is necessary to sequence the whole genome of *Armillaria* and analyse its genome to identify the genetic variation that is conducive to the establishment of a symbiotic relationship between *Armillaria* and *G. elata.* These results provide genomic information for studying the symbiotic mechanism between *Armillaria* and *G. elata* at the gene level and developing useful *Armillaria* strains to improve the yield of *G. elata*.

To date, several whole genome sequencing datasets of *Armillaria* have already been published, which revealing plant cell wall degradation enzymes and some secreted proteins [23–25]. Even among closely related *Armillaria* species, fungal mitogenomes are highly variable in size, gene content, and genome organization [26]. Mobile genetic elements invading introns and intergenic sequences of *Armillaria* mitogenomes play an important role in shaping their genome structure [26]. Mobile and highly repetitive elements (REs) play a significant role in the replication and formation of nucleoprotein complexes and affect the expression of genes [27]. Most REs are derived from transposable elements (TEs) [27]. Fungal genomes are densely packed with TEs [28, 29]. TEs may play important roles in the adaptation of fungi to new ecological niches [30]. In Magnaporthe oryzae, TE was found to be associated with the genes involved in host specialization [31]. However, there are few studies on the genome of *Armillaria* symbiotic with *G. elata*. Zhan found that there were approximately 23.6% repetitive sequences in the genome of a diploid *A. gallica* strain isolated from the tuber of *G. elata*, but most of the repetitive sequences were unknown [32]. The molecular mechanism of the symbiotic relationship between *Armillaria* and *G. elata* is still unclear. Therefore, genomic data are critical to resolve the complex properties of *Armillaria* species and to better study the symbiotic relationship between *Armillaria* and *G. elata*. It is necessary to study the genome of *Armillaria* species to clarify the characteristics of *Armillaria* and the symbiotic mechanism between *Armillaria* and *G. elata*.

In this study, we used PacBio Sequel and Illumina NovaSeq PE150 to analyse the whole genome sequence of the haploid *A. gallica* Jzi34 strain, which symbiotic with *G. elata*, and to assess specific and extended gene families. The results will provide enhanced insights for solving the complex characteristics of *Armillaria* species and help to study the symbiotic mechanism between *Armillaria* and *G. elata*.

## Results

### Genome assembly and characteristics analysis

The *A. gallica* Jzi34 genome assembly contained ~ 79.9 Mbp. The final assembly results showed that the genome assembly consisted of 60 contigs with an N50 of 2,535,910 bp. The maximum length of the assembled contig was 8,033,447 bp. The total contig length was 79,897,101 bp. The average GC content of the resulting *A. gallica* Jzi34 genome was 47.44% (Table 1).

BUSCO v3.0.2 software was used to assess the integrity of the genome assembly and annotation completeness. The results showed that 95.9% (278 BUSCOs) were complete genes based on the BUSCO assessment, while 1.4% (4 BUSCOs) were fragmented and 2.7% (8 BUSCOs) were missing (Table 1).

**Table 1:** Genome assembly parameters of *A. gallica* Jzi34, *A. gallica* 012m, *A. gallica* Ar21-2, *A. cepistipes* B5, *A. ostoyae* C18/9, *A. solidipes* 28 − 4, *A. mellea* DSM 3731, and *A. fuscipes* CMW2740 [23–25, 32]

Parameter	*A. gallica* Jzi34	*A. gallica* 012m	*A. gallica* Ar21-2	*A. cepistipes* B5	*A. ostoyae* C18/9	*A. solidipes* 28 − 4	*A. mellea* DSM 3731	*A. fuscipes* CMW2740
Scaffolds	60	63	319	287	106	229	29,300	24,403
Contigs	68	63	1866	740	106	848	65,823	27,509
Maximum length, bp	8,033,447	6,431,929	4,779,317	6,135,745	6,405,655	3,399,694	639,705	157,180
Scaffold size, bp	79,897,101	87,305,441	85,336,812	75,828,441	60,106,801	58,009,494	79,545,241	52,984,320
Contig size, bp	80,196,756	87,305,441	78,368,969	75,822,108	60,106,801	55,743,814	67,014,010	52,475,986
Scaffold N50, bp	2,535,910	2,159,699	1,035,263	3,291,351	2,283,935	715,667	24,647	5,422
Contig N50, bp	2,534,025	2,159,699	146,437	655,924	2,283,935	242,961	3268	4836
GC (%)	47.44	47.38	47.35	47.71	48.33	48.35	47.26	47.68
Gene number (#)	16,280	26,261	25,704	23,461	22,705	20,811	14,473	14,515
Complete BUSCOs	95.9%	95.8%	98.6%	95.1%	95.6%	98.4%		
Duplicated BUSCOs	1.4%	4.9%	3.8%	2.9%	2.2%	3.1%		
Fragmented BUSCOs	1.4%	3.3%	1.0%	4.0%	3.7%	1.3%		
Missing BUSCOs	2.7%	0.9%	0.4%	0.9%	0.7%	0.3%		

### Gene Prediction

Functional annotation revealed a total of 16,280 protein coding genes with an average gene length of 1,258 bp in the *A. gallica* Jzi34 genome (Table 1). These genes, with a total length of 20,474,301 bp, accounted for 25.63% of the total genome length. Furthermore, 14,044 RE sequences were identified in the *A. gallica* Jzi34 genome assembly. Dispersed repeat sequences (6,505) were classified into six groups, namely, LTR, DNA, LINE, SINE, RC, and unknown and their average lengths were 550, 92, 77, 70, 436 and 59 bp, respectively (Table 2). The LTR was widely represented in *A. gallica* Jzi34, accounting for 3.1105% of the total genome. A total of 7,539 tandem repeat sequences (TRs) were classified into TR (4,127), Minisatellite DNA (3,118), and Microsatellite DNA (294); they were recorded for 0.3926%, 0.1814% and 0.0137% of the total genome with repeat sizes of 1 ~ 1,891 bp, 10 ~ 60 bp, and 2 ~ 6 bp, respectively (Table 3). The RNAs in *A. gallica* Jzi34 are summarized in Table 4. The number of tRNAs (308) was highest, followed by snRNAs (24).

**Table 2:** The dispersed repeat sequences (DRs) of *A. gallica* Jzi34 genome on class

Type	Number (#)	Total Length(bp)	In Genome (%)	Average length(bp)
LTR	4,624	2,485,178	3.1105	550
DNA	751	68,522	0.0858	92
LINE	594	43,237	0.0541	77
SINE	14	975	0.0012	70
RC	511	207,302	0.2595	436
Unknown	11	644	0.0008	59
Total	6,505	2,802,758	3.508	443

**Table 3:** Tandem repeat sequences (TRs) of *A. gallica* Jzi34 genome on class

Type	Number(#)	Repeat Size(bp)	Total Length(bp)	In Genome(%)
TR	4,127	1 ~ 1,891	313,651	0.3926
Minisatellite DNA	3,118	10 ~ 60	144,916	0.1814
Microsatellite DNA	294	2 ~ 6	10,937	0.0137

**Table 4:** *A. gallica* Jzi34 genome data related RNAs

Type	Number(#)	Average length(bp)	Total length(bp)
tRNA	308	90	27,846
5s(denovo)	4	114	456
5.8s(denovo)	0	0	0
18s(denovo)	2	1,804	3,607
28s(denovo)	2	5,744	11,487
sRNA	0	0	0
snRNA	24	124	2,984
miRNA	0	0	0

### Gene Annotation

**Fig. 1:**
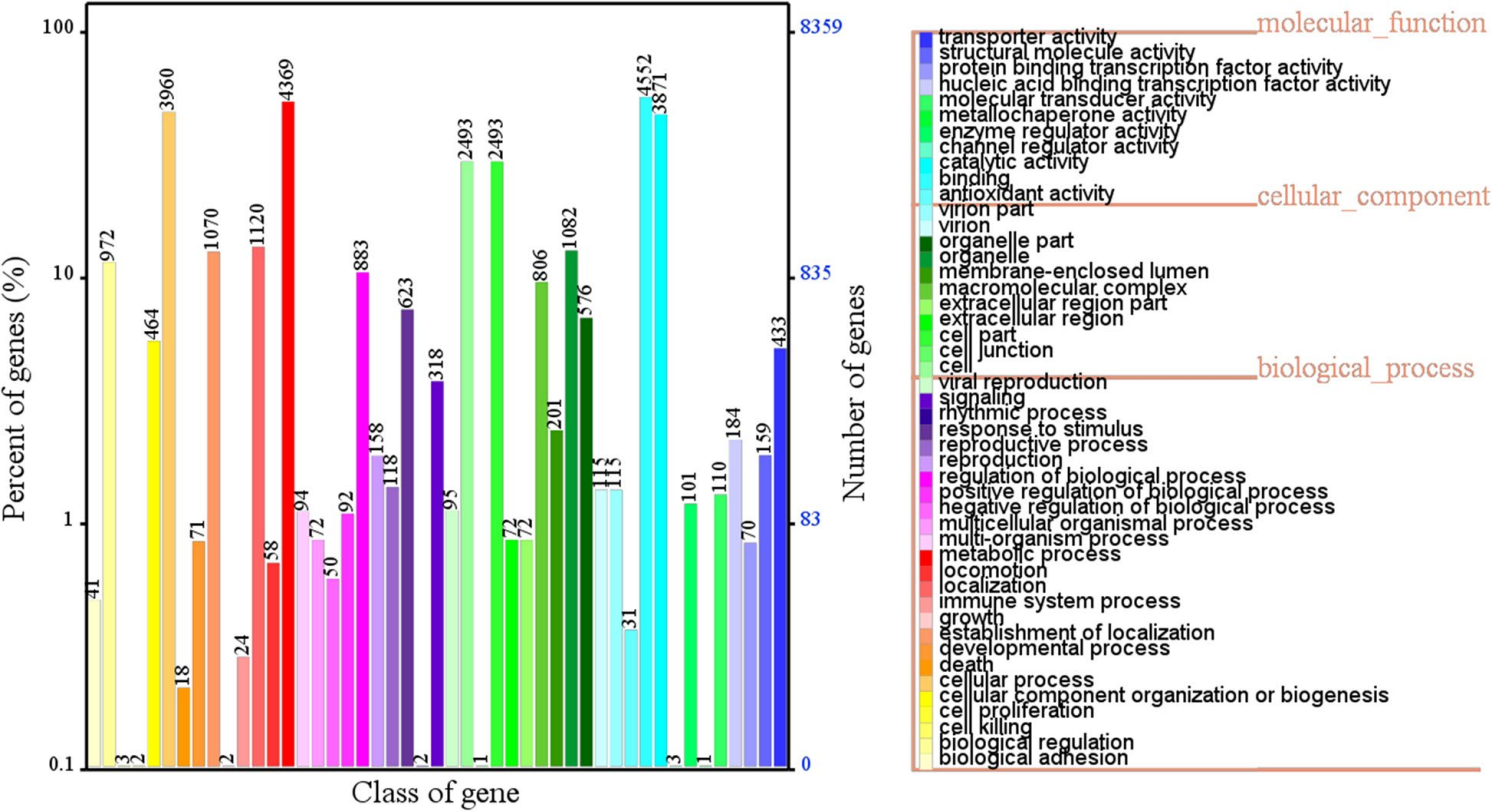
GO functional annotation of the *A. gallica* Jzi34 genome. GO annotation is divided into three major categories and 47 subclasses. A different colour represents each subclass. The x-axis represents the class of genes and the y-axis represents the percent of genes (%). The z-axis represents the number of genes

In the GO analysis, 8,358 coding genes were annotated in *A. gallica*; and classified into molecular function, cellular component, and biological process (Fig. 1). Among these categories, six functional entries had more than 2,400 annotated genes. These genes were significantly enriched in binding (4,552), metabolic process (4,369), cellular process (3,960), catalytic activity (3,871), cell (2,493) and cell part (2,493).

**Fig. 2:**
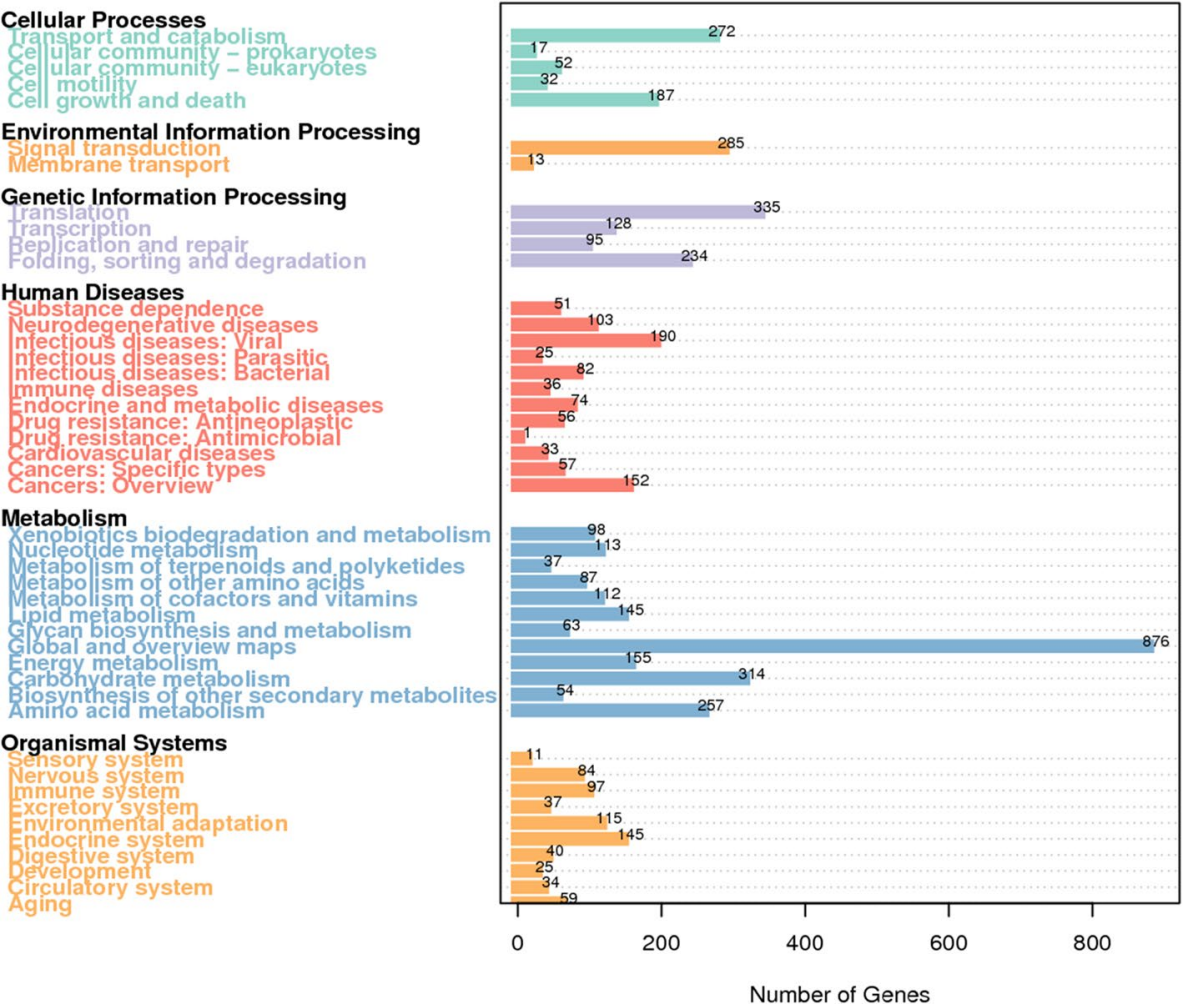
KEGG pathway annotation of the *A. gallica* Jzi34 genome. KEGG pathway annotation is divided into six major classes and 45 subclasses. The x-axis indicates the gene number of the concerned subclass. Each subclass is represented by a different colour

To further reveal the biological function of these coding genes in *A. gallica*, KEGG pathway annotation was performed. The KEGG database annotated 9,757 genes. The biological pathways are classified into six categories. Among the categories, metabolic pathways had the greatest number of annotated genes (2,311), with a high number of genes for carbohydrate metabolism and amino acid metabolism (Fig. 2).

**Fig. 3:**
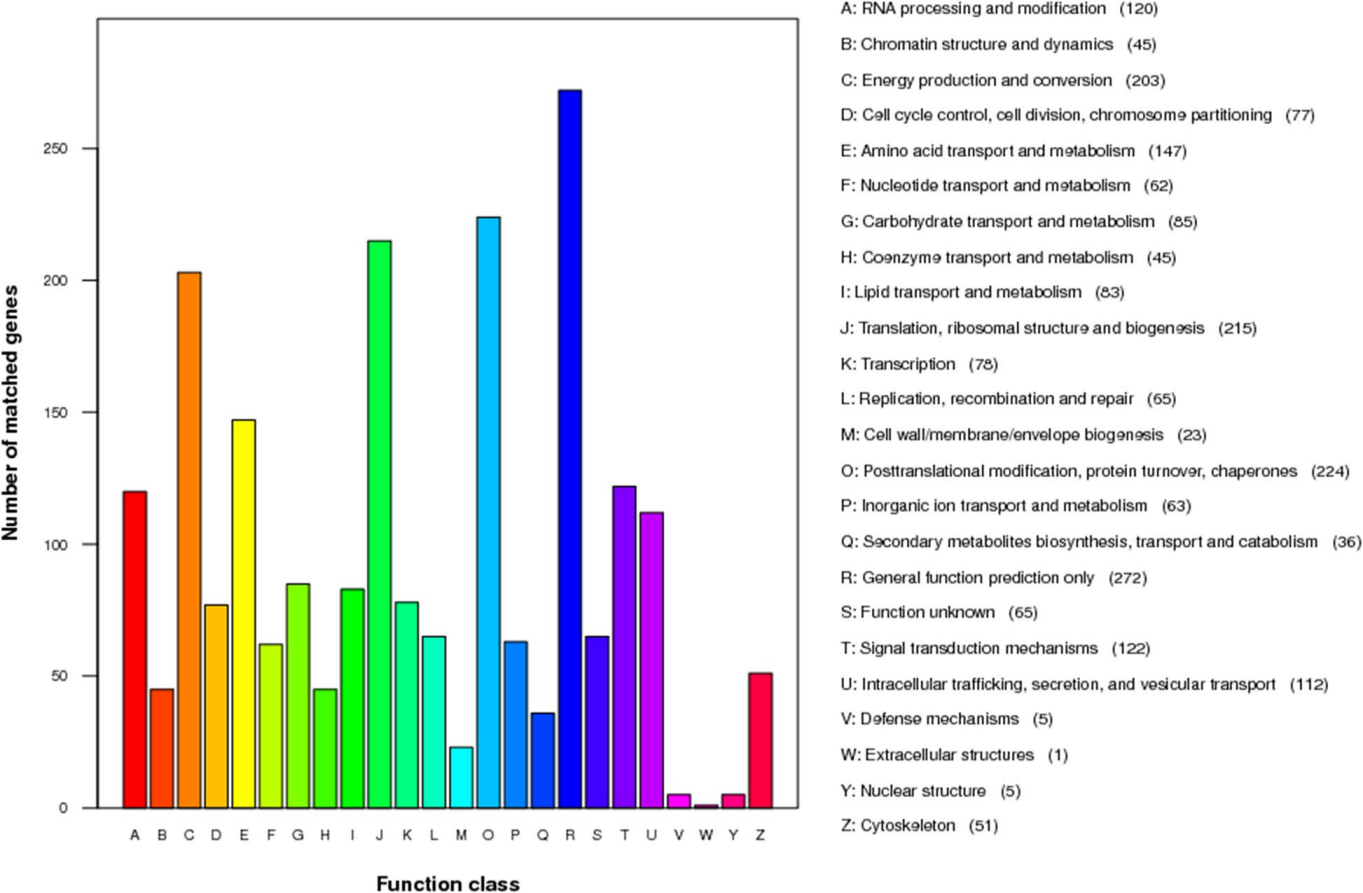
KOG functional annotation of proteins in the *A. gallica* Jzi34 genome. KOG function classification is summarized in 26 classes. The x-axis indicates each class and the y-axis shows the number of matched genes. The names of groups and number of genes are mentioned

Based on KOG annotation, 1,982 genes of functional categories were annotated. The protein functions were mainly enriched in general function prediction only (272); post-translation modification, protein turnover, chaperones (224); translation, ribosomal structure and biology (215); energy production and conversion (203); amino acid transport and metabolism (147); and other aspects (Fig. 3).

### The Carbohydrate Enzyme Classification and Annotation

**Fig. 4:**
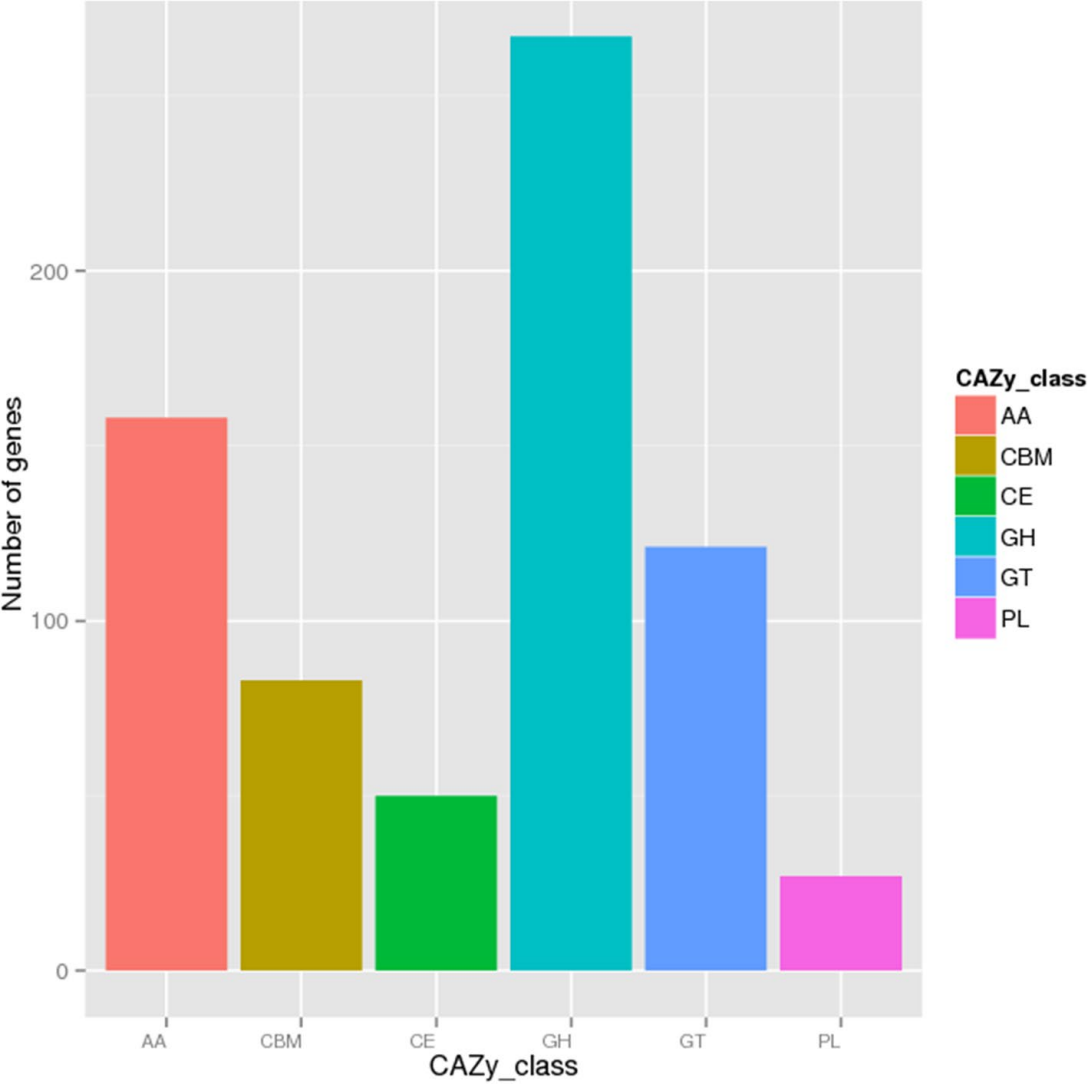
Carbohydrate enzyme functional classification. The CAZy classification is divided into 6 classes, including AA, CBM, CE, GH, GT and PL. The x-axis shows the CAZy class and the y-axis indicates the number of genes

A total of 664 CAZymes were annotated in the genome of *A. gallica* Jzi34, which included 267 glycoside hydrolases (GHs), 158 auxiliary activity enzymes (AAs), 121 glycosyl transferases (GTs), 83 carbohydrate-binding modules (CBMs), 50 carbohydrate esters (CEs), and 27 polysaccharide lyases (PLs) (Fig. 4). Compared with the genome assembly of other *Armillaria*, *A. gallica* Jzi34 had fewer CAZymes, while it had the largest set of GTs involved in glycosylation and the synthesis of polysaccharides (Table 5).

Although *A. gallica* Jzi34 had fewer AAs, its AA3-2 subfamilies (77) are more abundant than those of *A. gallica* 012m (45) and *A. gallica* Ar21-2 (58). The copy number of the AA3-2 subfamily was also higher than those of *A. cepistipes* B5, *A. ostoyae* C18/9, and *A. solidipes* 28 − 4 (Table 5).

**Table 5:** CAZymes and Cytochrome P450 of *A*. *gallica* Jzi34, *A. gallica* 012m, *A. gallica* Ar21-2, *A. ostoyae* C18/9, *A. solidipes* 28 − 4, *A. cepistipes* B5 [25, 32]

Description	Family	Subfamily	*A. gallica* Jzi34	*A. gallica 012m*	*A. gallica* Ar21-2	*A. ostoyae C18/9*	*A. solidipes* 28 − 4	*A. cepistipes* B5
CAZymes	Total		664	824	826	764	789	810
	GH		267	331	318	300	302	329
	GT		121	100	106	92	93	97
	PL		27	25	25	31	29	27
	CE		50	142	144	135	150	136
	CBM		83	82	79	71	79	66
	AA		158	170	178	156	158	181
		AA3_2	77	45	58	46	51	64
Cytochrome P450			349	271	330	271	279	324

### P450

**Fig. 5:**
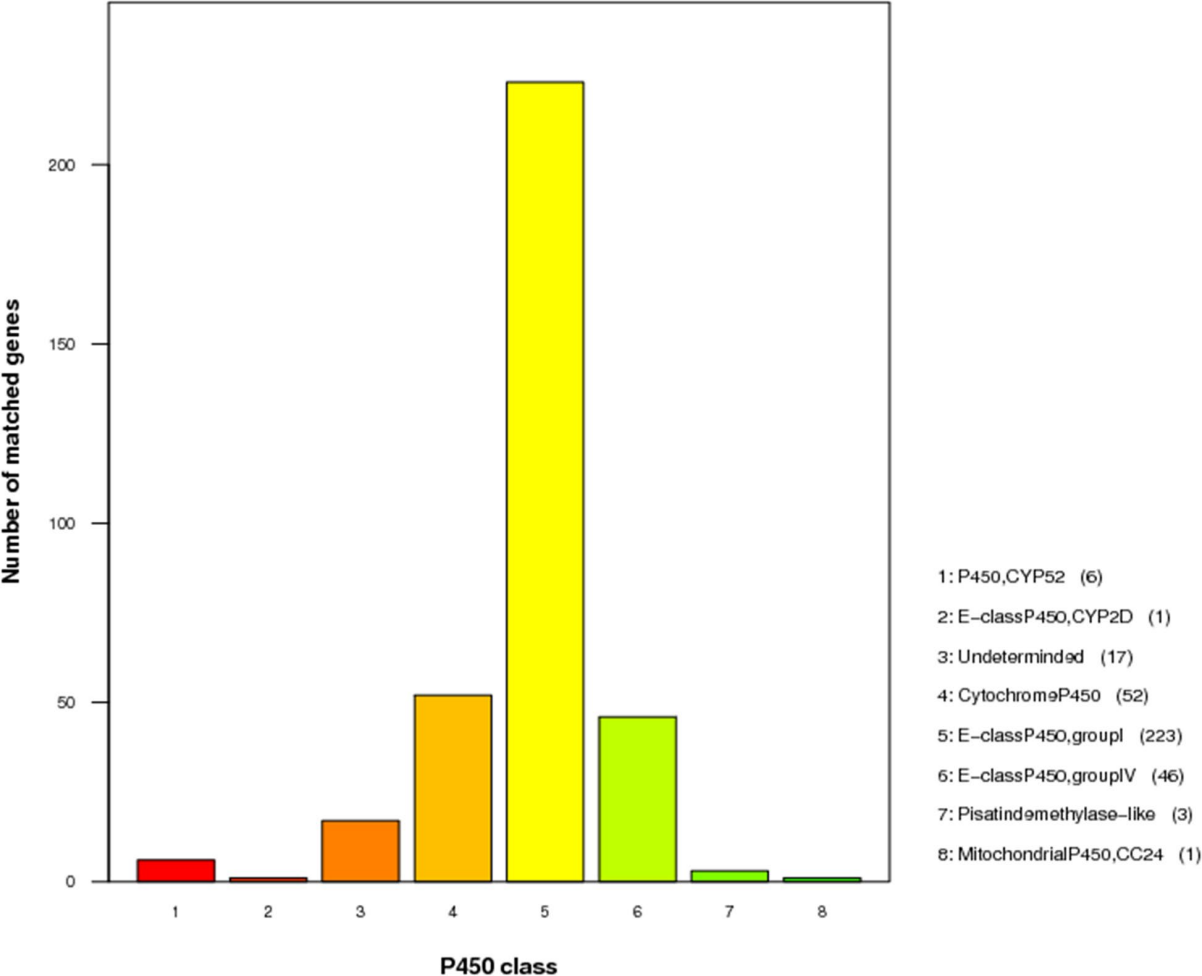
Functional classification of cytochrome P450 in the *A. gallica* Jzi34 genome. cytochrome P450 annotation is divided into 12 classes. The x-axis represents the P450 class. The y-axis represents the number of matched genes

P450 enzymes are involved in the construction of important metabolites in fungi, but also play a key role in adaptation to specific environments by modifying different compounds. In total, 349 P450 genes were identified in *A. gallica* Jzi34. The number was greater than those of *A. gallica* Ar21-2 (330), *A. cepistipes* B5 (324), *A. solidipes* 28 − 4 (279), *A. ostoyae* C18/9 (271), and *A. gallica* 012m (271) (Table 5). According to the annotation of these genes in the P450 database, they were divided into 8 groups. Most (223) of the genes were predicted to encode “E-class P450, group I”, followed by “Cytochrome P450” (52) and “E − classP450, group IV” (46) (Fig. 5).

### Pathogen Host Interaction (PHI)

**Fig. 6:**
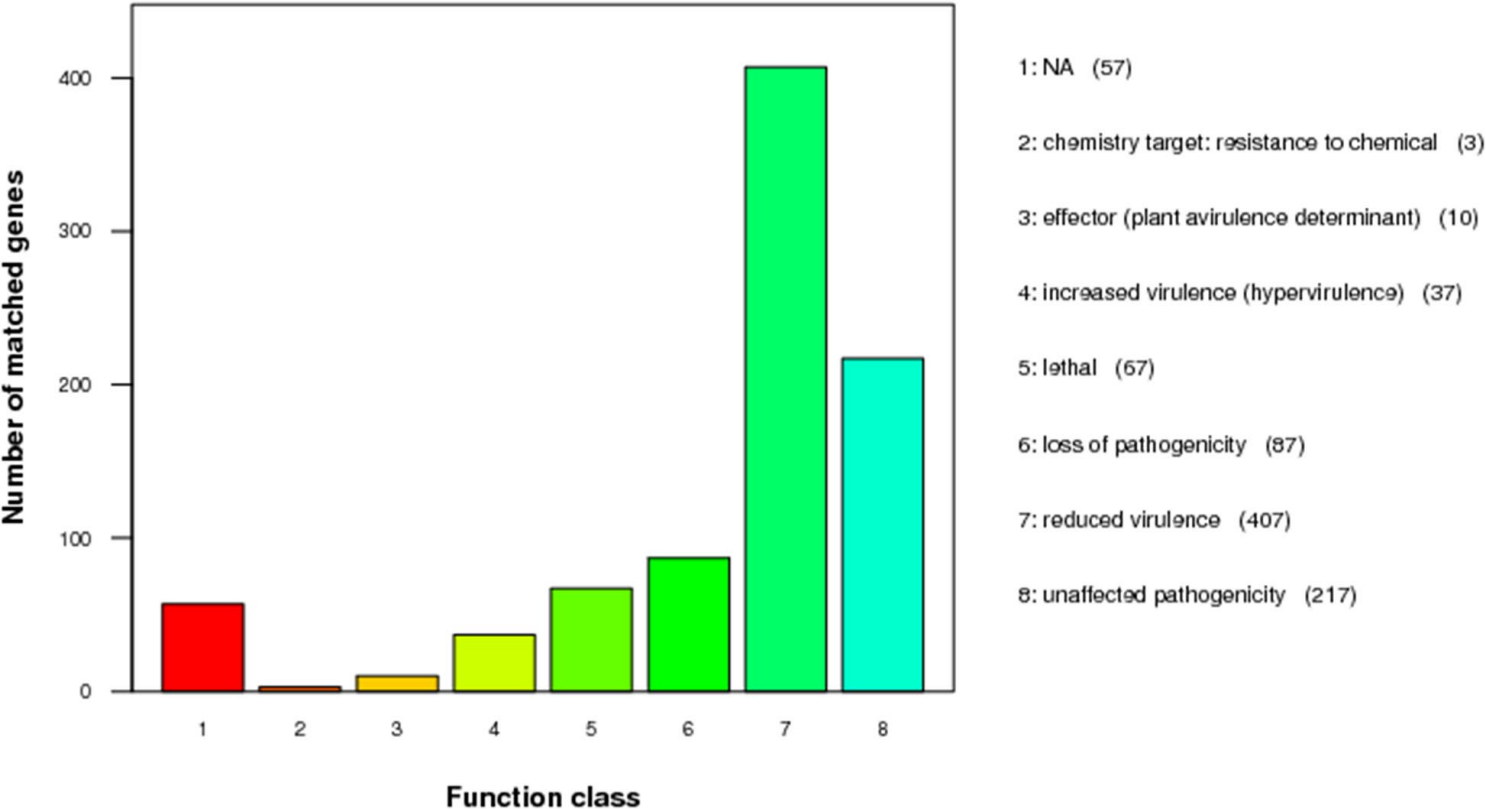
PHI classification in the *A. gallica* Jzi34 genome. PHI annotation is classified into 8 classes. The x -axis shows each class, and the y-axis represents the number of matched genes

A total of 885 genes were annotated by searching the PHI database. The genes for pathogenicity of *A. gallica* Jzi34 in the pathogen host interaction (PHI) database were annotated to 8 categories. It revealed that the largest number (407) of the genes was associated with the functional class of “reduced virulence”, followed by the functional class of “unaffected pathogenicity” (217) and “loss of pathogenicity” (87). It also found that 67, 37 and 10 genes were identified as “lethal function”, “increased virulence” and “effector”, respectively. In addition, 57 genes were found to be involved in the NA function class (Fig. 6).

### Synteny analysis of P450 genes

**Fig. 7:**
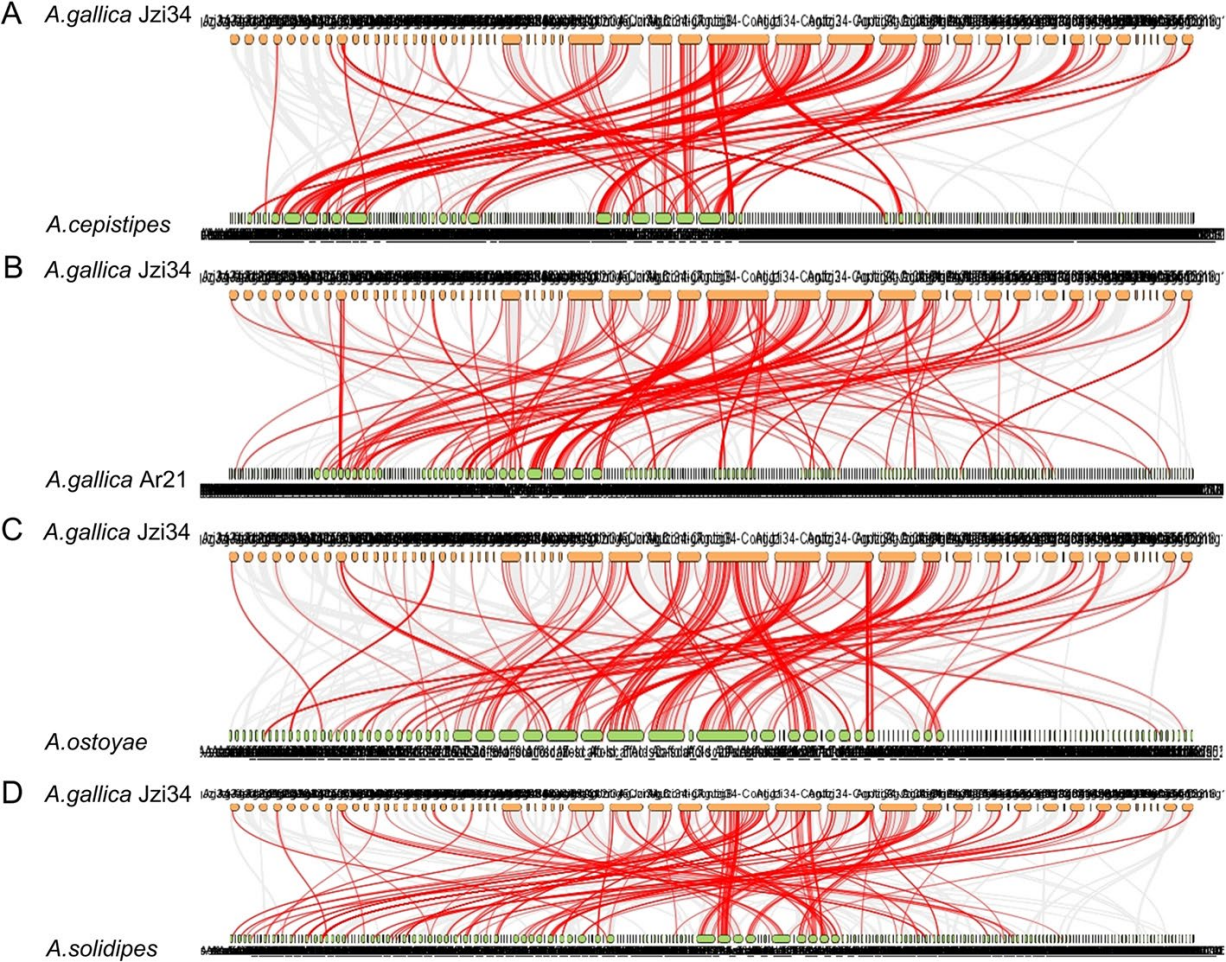
Syntenic analysis of P450 genes between *A.gallica* Jzi34 and four other *Armillaria*. The gray lines at the bottom indicate the collinear blocks within *A.gallica*Jzi34 and other *Armillaria* genomes. The red lines indicate the pairs of P450 genes. The results of the syntenic analysis between *A.gallica* Jzi34 and other *Armillaria*, including *A. cepistipes* B5, *A. gallica*Ar21-2, *A. ostoyae* C18/9, and* A. solidipes* 28-4 (**A**–**D**)

To further explore the evolutionary relationship of P450 proteins in *A. gallica* Jzi34 (AgP450s) and other *Armillaria*, the syntenic relationship was traced between AgP450s and homologs in other *Armillaria* species. The number of homolog pairs between AgP450s and P450s in the other *Armillaria*, including *A. gallica* Ar21-2, *A. cepistipes* B5, *A. solidipes* 28 − 4, and *A. ostoyae* C18/9, was 248, 263, 215, and 226. *A. gallica* Jzi34 had many specific P450 genes (Table 6). The results showed that the genetic relationship between AgP450s and P450s in *A. gallica* Ar21-2, *A. cepistipes* B5, *A. solidipes* 28 − 4, and *A. ostoyae* C18/9 was complex (Fig. 7A-D).

**Table 6:** Homologous gene pairs between AgP450s and P450s in the other *Armillaria*

*Armillaria* species	Homolog pairs	None pairs
*A.gallica* Jzi34 vs *A.cepistip* B5	263	86
*A.gallica* Jzi34 vs *A.gallica* Ar21-2	248	101
*A.gallica* Jzi34 vs *A.ostoyae* C18/9	226	123
*A.gallica* Jzi34 vs *A.solidipes* 28 − 4	215	134

Note: There were 349 AgP450s in *A.gallica* Jzi34. Homolog pairs: The number of AgP450 that had homologous gene in other *Armillaria*. None pairs: The number of AgP450 that had none homolog in other *Armillaria*

To better understand the evolutionary constraints affecting the P450 gene family, the Ka / Ks ratios of P450 gene pairs were calculated. The Ka / Ks ratio of the gene pairs between AgP450 genes and the P450 orthologous genes was less than 1, which suggests that the P450 gene family have undergone a strong selective purge pressure during evolution (Additional file 1 Table S 1).

## Discussion

The genome size of *A. gallica* Jzi34 (~ 79.9 Mbp) was within the genome size range of other *Armillaria* species. The five draft genomes of *Armillaria* were assembled into 63–29,300 scaffolds comprising 53–87 M [25, 32]. However, the genomes of *A. gallica* Ar21-2 and *A. solidipes* 28 − 4, as pathogens of wood, contain many ambiguous bases [25, 32]. In this study, we obtained a 79.9 Mbp genome of *A. gallica* Jzi34, which consisted of 60 contigs with an N50 of 2,535,910 bp and 47.44% GC content. A total of 16,280 genes were identified in the *A. gallica* Jzi34 genome, with an average length of 1258 bp (Table 1). This was less than the number of genes in closely related species, which contained 14,473–26,261 genes [23, 25, 32]. However, even among closely related *Armillara* species, fungal mitogenomes are significantly different in size, gene content, and genome organization [26].

Compared with the complete BUSCO genes in *A. gallica* Ar21-2 (98.6%) as wood pathogens, the lower genome completeness of *A. gallica* 012m (95.8%) and *A. gallica* Jzi34 (95.9%) established a symbiotic relationship with *G. elata* (Table 1). The genome completeness of *A. gallica* Jzi34 was lower than that of *A. gallica* Ar21-2. This may be related to the extensive loss of lignin and cellulose degrading enzymes involved in the transition from saprophytic to symbiotic [33].

In this study, only 4.1% of RE sequences were identified in the *A. gallica* Jzi34 genome assembly, but few sequences were unknown (Table 2). The genome of *A. gallica* strain 012m had approximately 23.6% repetitive sequences, but most of the repetitive sequences were unknown [32]. There was a significant difference in the number of TEs among different *Armillaria* species [25, 33]. Moreover, LTR elements of the identified TE classes were the most frequent in basidiomycete fungi [34]. The LTR (6,505) was also widely represented in the *A. gallica* Jzi34 genome, accounting for 3.1% of the *A. gallica* Jzi34 genome (Table 2).

*A. gallica* Jzi34 had fewer CAZymes than other *Armillaria* species. This finding coincided with previous research showing that certain CAZyme families contracted during the evolution of mycorrhizal fungi compared with those of their saprotrophic ancestors [35, 36]. Although the AA family was significantly contracted in *A. gallica* Jzi34, its AA3-2 subfamily had more genes than the other *Armillaria* (Table 5). Moreover, compared with the other *Armillaria* genomes, *A. gallica* Jzi34 had the largest set of GTs. The AA3-2 subfamily has been considered important enzymes in the biodegradation of lignocellulose [37, 38]. Glycosylation catalysed by glycosyltransferases (GTs) contributes to fungal infection and secondary metabolic synthesis. GTs are critical for fungal growth, host penetration and immune evasion [39, 40]. These may be beneficial for *A. gallica* Jzi34 to establish a symbiotic relationship with *G. elata*, and continue to provide nutrition for *G. elata*.

In fungi, P450 enzymes are widely involved in a variety of physiological reactions, contributing to the adaptation and fecundity of fungi in specific ecological niches [41, 42]. P450 enzymes may play an important role in fungal colonization of plant material [43]. Filamentous fungi symbionts are important in detoxifying host chemical defence compounds [44]. There were more P450 genes identified in *A. gallica* Jzi34 (349) than in *A. gallica* Ar21-2 (330), *A. cepistipes* B5 (324), *A. solidipes* 28 − 4 (279), *A. ostoyae* C18/9 (271), and *A. gallica* 012m (271) (Table 5). Therefore, an increase in the P450 genes may contribute to the growth of *A. gallica* Jzi34 in a symbiotic relationship with *G. elata*.

The syntenic analysis on AgP450s and the homologs showed that more P450 orthologous pairs were found between *A. gallica* Jzi34 and two other *Armillaria*, including *A. gallica* Ar21-2 and *A. cepistipes* B5. However, the homologous gene pairs between *A. gallica* Jzi34 and *A. solidipes* 28 − 4 and the homologous P450 genes pairs between *A. gallica* Jzi34 and *A. ostoyae* C18/9 were relatively less (Table 6). These results are consistent with the phylogenetic relationship between *A. gallica* and the other *Armillaria* [25, 32]. There were still many AgP450s in *A. gallica* Jzi34 did not exhibit the homologous gene pairs with these *Armillaria*, including *A. gallica* Ar21-2, *A. cepistipes* B5, *A. solidipes* 28 − 4, and *A. ostoyae* C18/9 (Table 6). This may be related to the expansion of the cytochrome p450 family in *Armillaria* species [25], suggesting the unique role in biology of these fungi. It may also be related to the symbiosis of *A. gallica* Jzi34 and *G. elata*.

## Conclusions

In this study, we sequenced the genome and annotated the functional genes of the *A. gallica* Jzi34 strain, which was symbiotic with *G. elata*. A total of 16,280 genes were predicted in the genome. Repetitive sequences represent approximately 4.1% of the genome. Genome comparison analyses with other *Armillaria* strains showed that carbohydrate enzyme genes were significantly contracted in *A. gallica* Jzi34. However, it had an expansion of cytochrome P450 and AA3-2 subfamily genes. The synteny analysis result of P450 genes showed that the evolutionary relationship of P450 proteins in *A. gallica* Jzi34, *A. gallica* Ar21-2, *A. cepistipes* B5, *A. solidipes* 28 − 4, and *A. ostoyae* C18/9 is complex. The study elucidates the characteristics of *A. gallica* Jzi34. In addition, it provides an important genomic resource for further studies on the symbiotic relationship between *Armillaria* and *G. elata*.

## Methods

### Materials and sequencing

The haploid *A. gallica* Jzi34 strain used for genome sequencing was isolated from a fruiting body as a single spore isolate. The fruiting body was collected from a plantation field in Luquan, Kunming, Yunnan Province, China, in 2021. For genomic DNA, *A. gallica* Jzi34 was grown in a liquid culture medium [45]. Then, the culture was shaken for 10 days at 115 rpm/min and 25°C. Mycelium was collected and frozen in liquid nitrogen. Genomic DNA was extracted with the SDS method [46]. The whole genome of *A. gallica* Jzi34 was sequenced using the PacBio Sequel platform and Illumina NovaSeq PE150.

### Genome assembly

SMRT Link v5.0.1 was used to accomplish preliminary assembly [47, 48]. To ensure the accuracy of the subsequent analysis results, the low-quality reads were filtered (less than 500 bp) to obtain clean data. Using the automatic error correction function of the SMRT portal, the long reads were selected (more than 6000 bp) as the seed sequence, and the other shorter reads were aligned to the seed sequence by Blasr, so as to improve the accuracy of the seed sequences. After assembly, we obtained an initial result. The Variant Caller module of SMRT Link software was used to correct and count the variant sites in the preliminary assembly results.

We used BUSCO Version 3.0.2 to assess the completeness of the assemblies. The lineage dataset is: fungi_odb9 (Creation date: 2016-02-13, number of species: 85, number of BUSCOs: 290).

**Genome component prediction** included the prediction of the coding gene, repetitive sequences and noncoding RNA. The steps were as follows:

1. The Augustus 2.7 program was used to retrieve the related coding genes.
2. The interspersed repetitive sequences were predicted using RepeatMasker (http://www.repeatmasker.org/) [49]. The tandem repeats were analysed by the Tandem Repeats Finder [50].
3. Transfer RNA (tRNA) genes were predicted by tRNAscan-SE [51]. Ribosome RNA (rRNA) genes were analysed by rRNAmmer [52]. sRNA, snRNA and miRNA were predicted by BLAST against the Rfam database (default parameters).

### Gene function

We used seven databases to predict gene functions. They were respective GO (Gene Ontology) [53], KEGG (Kyoto Encyclopedia of Genes and Genomes) [54, 55], KOG (Clusters of Orthologous Groups), NR (Non-Redundant Protein Database) [56], TCDB (Transporter Classification Database), P450 [57, 58], and, Swiss-Prot. A whole genome BLAST search (E-value less than 1e-5, minimal alignment length percentage larger than 40%) was performed against the above seven databases. The secretory proteins were predicted by the Signal P database. For pathogenic fungi, we added the pathogenicity analyses. PHI (pathogen host interactions) was used to perform the above analyses [59, 60]. Carbohydrate-active enzymes were predicted by the Carbohydrate-Active Enzymes Database.

### Syntenic analysis and Ka/Ks analysis

The four *Armillaria* species amino acid, genome, and CDS sequence assembly and corresponding annotation were downloaded from the US DoE JGI fungal genomics resource database (https://mycocosm.jgi.doe.gov/) [25]. TBtools [61] was used to analyse the homology and collinearity of the P450 family gene between *A. gallica* Jzi34 and the other *Armillaria* (default parameters).

## Supplementary Information

**Additional file 1: TableS1.** Ka/Ks value for the collinear P450 gene pairs between *A. gallica* Jzi34 and other *Armillaria*.

## Data Availability

This Whole Genome Shotgun project has been deposited at DDBJ/ENA/GenBank under the accession JANYMC000000000. The version described in this paper is version JANYMC010000000. The bioproject is PRJNA874901. The BioSample is SAMN30589529. (http://www.ncbi.nlm.nih.gov/bioproject/874901)

## Abbreviations

AA: Auxiliary activity
BLAST: Basic Local Alignment Search Tool
bp: Base pair
CBM: Carbohydrate-binding modules
CE: Carbohydrate esters
DNA: Deoxyribonucleic acid
GH: Glycoside hydrolase
GT: Glycosyl transferase
LINE: Long interspersed nuclear element
LTR: Long terminal repeat
Mbp: Million base pair
N50: A weighted median statistic such that 50% of the entire assembly is contained in contigs or scaffolds equal to or larger than this value
PL: Polysaccharide lyase
PHI: Pathogen host interaction
RC: Rolling coil
RE: Repetitive element
RNA: Ribonucleic acid
rRNA: ribosomal RNA
SINE: Short interspersed nuclear element
TE: Transposable element
TR: Tandem repeat sequences
